# Multiple nucleic acid cleavage modes in divergent type III CRISPR systems

**DOI:** 10.1093/nar/gkw020

**Published:** 2016-01-21

**Authors:** Jing Zhang, Shirley Graham, Agnes Tello, Huanting Liu, Malcolm F. White

**Affiliations:** Biomedical Sciences Research Complex, University of St Andrews, Fife KY16 9ST, UK

## Abstract

CRISPR-Cas is an RNA-guided adaptive immune system that protects bacteria and archaea from invading nucleic acids. Type III systems (Cmr, Csm) have been shown to cleave RNA targets *in vitro* and some are capable of transcription-dependent DNA targeting. The crenarchaeon *Sulfolobus solfataricus* has two divergent subtypes of the type III system (Sso-IIID and a Cmr7-containing variant of Sso-IIIB). Here, we report that both the Sso-IIID and Sso-IIIB complexes cleave cognate RNA targets with a ruler mechanism and 6 or 12 nt spacing that relates to the organization of the Cas7 backbone. This backbone-mediated cleavage activity thus appears universal for the type III systems. The Sso-IIIB complex is also known to possess a distinct ‘UA’ cleavage mode. The predominant activity observed *in vitro* depends on the relative molar concentration of protein and target RNA. The Sso-IIID complex can cleave plasmid DNA targets *in* vitro, generating linear DNA products with an activity that is dependent on both the cyclase and HD nuclease domains of the Cas10 subunit, suggesting a role for both nuclease active sites in the degradation of double-stranded DNA targets.

## INTRODUCTION

One mechanism used by bacteria and archaea to protect themselves from invading nucleic acids is the adaptive immune system CRISPR-Cas, comprised of CRISPR arrays of repeat-spacer units and CRISPR-associated (Cas) genes (reviewed in (1)). The CRISPR-Cas system acts through three stages. First, short fragments of invading viruses and plasmids are captured and integrated into CRISPR arrays in a process known as acquisition or adaptation (2), generating a library of ‘non-self’ spacer sequences. Second, the CRISPR loci are transcribed and processed into short CRISPR RNAs (crRNAs) (reviewed in (3)). These unit length crRNAs are subsequently assembled with Cas proteins into large ribonucleoprotein effector or surveillance complexes. Guided by the crRNA sequence, the effector complexes detect and degrade cognate nucleic acids (reviewed in (4)).

CRISPR-Cas systems have been classified into five types (5,6). Distant phylogenetic relationships have been reported between several proteins from type I and III systems (7), in particular the Cas7 subunit (8). This relationship was confirmed by electron microscopy studies of the type III complexes, which revealed a helical backbone formed by Cas7 family subunits binding to the crRNA (9–11), with a striking similarity to that observed in the type I Cascade structure (12). However, these two systems are mechanistically distinct. Type I systems target double-stranded DNA and recruit the Cas3 helicase:nuclease protein to unwind and degrade target DNA. In contrast, type III systems were originally shown to target single-stranded RNA using either a ruler-dependent cleavage with 6 nucleotide (nt) spacing, as observed for the type IIIB complex from *Pyrococcus furiosus* (13) or a sequence-dependent ‘UA’ cleavage mechanism for the Cmr7-containing type IIIB complex from *Sulfolobus solfataricus* (14). These activities have also been shown to occur *in vivo* in two *Sulfolobus* species (15,16).

In contrast, the type IIIA systems have long been known to possess a DNA targeting activity *in vivo* (17), but this proved difficult to demonstrate *in vitro*, although the HD nuclease domain of the large subunit (Csm1/Cas10) was reported to possess DNA nuclease activity (18). Recently, a ruler-dependent ‘RNA shredding’ activity was described for the type IIIA complex from *Streptococcus thermophilus* (19). This study demonstrated that the bound RNA target was cleaved at regular 6 nt intervals along the Cas7 backbone of the complex, an activity dependent on a conserved acidic residue in the Csm3 subunit (19). Subsequently, this mechanism of RNA degradation has been observed for several other type III systems (20–23). Most recently, the crystal structure of a chimeric type IIIB complex bound to crRNA and target RNA revealed regular nt flipping of crRNA with a 6 nt periodicity, caused by the insertion of a conserved beta-hairpin ‘thumb’ of the Cmr4 (Cas7) subunit into the RNA duplex (24). The thumb includes the conserved acidic residue important for target RNA cleavage, suggesting that the local distortion and chemical environment at these positions is key to the periodic, backbone-mediated target RNA cleavage observed in the type III systems. This structural arrangement is strikingly reminiscent of the Cas7-mediated distortion observed in the structure of *Escherichia coli* Cascade bound to a crRNA:DNA heteroduplex (25), emphasizing the fundamental conservation of nucleic acid binding across the type I and type III systems (26).

In parallel with these studies, genetic analyses of a *Sulfolobus islandicus* type III system suggested that the complex cleaved DNA in a transcription-dependent mechanism (27). This observation was confirmed by studies of the *Staphylococcus epidermidis* type IIIA system by the Marraffini lab, who showed that DNA targeting by the type IIIA system is dependent on the transcription of the DNA target—a mechanism that they proposed allows dormant lysogenic phage DNA in the genome to escape degradation (28). Most recently, this activity was reconstituted *in vitro*, demonstrating that DNA cleavage by the *S. epidermidis* type IIIA complex was dependent on transcription of the target DNA by RNA polymerase (29). The active site responsible for the DNA cleavage, which was observed on only one DNA strand, was identified in the conserved cyclase domain of the large subunit, Csm1 (29), consistent with recent biochemical studies of Csm1 in isolation (30).

Thus, there is an emerging consensus that type III systems have a backbone-mediated ‘RNA shredding’ activity (although the functional relevance of this activity remains uncertain) and some at least also possess a transcription-dependent DNA targeting activity. The type III systems present in *S. solfataricus* strain P2 are relatively unusual. The complex previously denoted as belonging to type IIIA is now classified as a type IIID system (6). The type IIIB complex was reported to have a more compact, ‘crab claw’ structure and has an extra subunit, Cmr7, found in only a few type IIIB systems (14). Here, we demonstrate that the *S. solfataricus* type IIID and IIIB systems (previously known as the Csm and Cmr complexes, hereafter Sso-IIID and Sso-IIIB) also possess a backbone-mediated RNA shredding activity *in vitro*, confirming that RNA shredding is a universal property of all type III systems. Furthermore, the Sso-IIID complex can cleave dsDNA targets *in vitro* and the Sso-IIIB system can switch between sequence-dependent and backbone-mediated cleavage modes, depending on the amount of RNA target available.

## MATERIALS AND METHODS

### Oligonucleotides for polymerase chain reaction (PCR), subcloning and site directed mutagenesis

The oligonucleotides were synthesized by Integrated DNA Technologies (IDT) and the sequences are available on request from the corresponding author.

### Expression and purification of tagged Sso-IIID and Sso-IIIB complexes from S. solfataricus

The Sso-IIID complex used in this study was tagged on the large subunit, Csm1/Sso1428. The Sso-IIIB complex was tagged on the subunit Sso1990. The gene encoding Sso1428 or Sso1990 was PCR amplified and cloned into pMZ1 (31), then subcloned into expression vector pSVA9 that expresses the relevant subunit with a C-terminal strep-His tag (32). The expression vector carrying the gene encoding Sso1428 or Sso1990 was transformed into the *S. solfataricus* PH1–16 expression strain. After transformation, cells were first cultivated in unselective Brock medium containing 0.1% (w/v) tryptone and 10 μg/ml uracil, and then transferred to selective media containing 0.2% (w/v) glucose and 0.1% (w/v) NZ-amine without uracil. Once the A_600_ nm reached 0.6, cells were transferred to expression media containing 0.2% (w/v) arabinose and 0.1% (w/v) NZ-amine to induce the expression of the tagged subunit and then collected at an A_600_ nm of 0.8 - 1.0. Cells were harvested by centrifugation and disrupted by sonication (Soniprep 150, MSE) in buffer A (20 mM HEPES pH 7.5, 250 mM NaCl, 30 mM imidazole). The supernatant was filtered and loaded onto a Histrap column (GE Healthcare) in buffer A. His-tagged proteins were eluted with a linear gradient of buffer B (20 mM HEPES pH 7.5, 250 mM NaCl, 500 mM imidazole). Fractions containing the tagged complex were pooled and exchanged into buffer C (20 mM Tris-HCl pH 8.0, 50 mM NaCl) then loaded onto a mono Q column (GE Healthcare) equilibrated in buffer C. Bound proteins were eluted with a linear gradient of buffer D (20 mM Tris-HCl pH 8.0, 1 M NaCl). Fractions containing the tagged complex were pooled, concentrated and loaded onto a Sephacryl 300 size exclusion column (GE Healthcare) equilibrated in buffer E (20 mM Tris-HCl pH 8.0, 250 mM NaCl). The purified proteins were concentrated and stored at 4°C.

To generate a variant Sso-IIIB complex with a mutated HD domain, the gene encoding the large subunit, Cmr2/Sso1991, was PCR amplified and cloned into a modified pMZ1 containing an N-terminal strep-His tag, resulting in pMZ1-Nhis-sso1991. Mutations of residues D181 and H182 to alanine were carried out by PCR mutagenesis using Phusion DNA polymerase (ThermoFisher Scientific) and pMZ1-Nhis-sso1991 as the template. Variants of the Sso-IIID complex with mutations targeted to the HD nuclease (H187A, D188A) and cyclase (D629A, D630A) domains were constructed in a similar manner. The procedures for subcloning, expression and purification of these variant proteins were as described above.

### Cloning, expression and purification of Sso-IIID subunits

The gene encoding each subunit except *sso1428* was amplified by PCR and cloned into an expression vector with an N-terminal 6x His-tag. The *sso1424* gene was cloned into the pEHisTev vector (33). The *sso1425*, *sso1426*, *sso1427*, *sso1430*, *sso1431* and *sso1432* genes were cloned into pDEST14 vector individually using the gateway recombination cloning (34). The constructs were expressed in C43 (DE3) *E. coli* grown in LB medium supplemented with appropriate antibiotics to an A_600nm_ of 0.6–0.8 at 37°C. Expression was induced by the addition of 0.4 mM IPTG and the cells were harvested after overnight incubation at 25°C. The cell pellets were resuspended in buffer F (50 mM Tris^.^HCl pH 7.5, 500 mM NaCl, 10 mM imidazole, 10% glycerol) and lysed by sonication. The lysate was clarified by centrifugation and filtered prior to loading onto a Histrap column (GE Healthcare) equilibrated in buffer G (50 mM Tris^.^HCl pH 7.5, 500 mM NaCl, 30 mM imidazole). The His-tagged proteins were eluted with a linear gradient of buffer H (50 mM Tris^.^HCl pH 7.5, 500 mM NaCl, 500 mM imidazole). Fractions containing the His-tagged protein were pooled and treated with TEV protease overnight at room temperature for cleavage of the his-tag. The cleaved protein was passed through a Histrap column in buffer G and collected in the flow through then further purified by size exclusion on a Superdex S200 column (GE Healthcare) equilibrated in buffer I (20 mM Tris^.^HCl pH 7.5, 250 mM NaCl). Fractions containing the proteins were concentrated and stored at −20°C.

The cell pellets for Sso1427 and Sso1431 were resuspended in buffer G containing 8 M urea and incubated overnight at room temperature. Samples were subject to sonication and ultracentrifugation at 40 000 rpm for 30 min prior to nickel affinity chromatography in buffer G containing 8 M urea. His-tagged proteins were eluted with a linear gradient of buffer H containing 8 M urea. Fractions containing the His-tagged proteins were stored at 4°C.

The *sso1428* gene was amplified and cloned into pMZ1 and then subcloned into pSVA9 and expressed from *S. solfataricus* as described above. The isolated Sso1428 subunit was purified using metal affinity, ion exchange, and size exclusion chromatography as for the intact complex (9). Fractions containing Sso1428 were concentrated and stored at 4°C.

### Purification and end-labelling of RNA oligonucleotides

RNA oligonucleotide substrates were supplied by IDT and purified and end labelled as described previously (14).

### Sso-IIID RNA cleavage assays

Purified Sso-IIID complex and 5′-^32^P end-labelled synthetic target RNA oligonucleotides were mixed in reaction buffer 1 (20 mM MES pH 6.0, 100 mM NaCl, 0.1 mg/ml BSA, 2 mM MgCl_2_) and incubated at 75°C for 20 min. The reaction was stopped by adding 1 μl of 20 mg/ml Proteinase K (Thermo scientific) and was further incubated at 37°C for 30 min. Then the reaction was phenol-chloroform (Ambion) extracted as described previously (35). Samples from the upper aqueous phase were mixed with formamide dye and heated at 95°C for 5 min before loading onto 20% polyacrylamide, 8 M urea, 1 x TBE gels. The gels were visualized by phosphor imaging. 5′ end-labelled RNA size standards (Decade Markers, Ambion) were used to determine the sizes of the observed products.

### Sso-IIIB RNA cleavage assays

Purified Sso-IIIB complex and unlabelled crRNA were mixed in reaction buffer 2 (20 mM MES pH 6.0, 100 mM potassium glutamate, 10 mM DTT, 10 mM MnCl_2_) and pre-incubated at 75°C for 10 min prior to the addition of ^32^P 5′ end-labelled synthetic target RNA to the reaction mix. The reaction was further incubated at 75°C for 10 min and processed as described above.

### Reconstitution of the Sso-IIID complex in vitro

Sso1424, Sso1425, Sso1426, Sso1427, Sso1428, Sso1430, Sso1431, Sso1432 and crRNA were mixed at a ratio of 3:1:4:1:1:1:1:1:1 in reaction buffer 1 and incubated at 45°C for 30 min prior to the addition of 4 nM radiolabelled target RNA. The reaction was further incubated at 75°C for 60 min then processed as described above.

### Sso-IIID plasmid cleavage assays

The pT6 plasmid (36) was used as the backbone for plasmid cleavage assays. A synthetic DNA fragment (gBlock, IDT) designed to incorporate sequences corresponding to three of the most abundant crRNA sequences identified in the Sso-IIID complex (corresponding to spacers A26 and B2) (9) and Sso-IIIB complex (spacer D63) (14) was subcloned into the BamHI site of the plasmid and the resultant plasmid, pSpacer, was checked by DNA sequencing. The sequence of the gBlock is presented in the supplementary data section. Purified wild type and mutant Sso-IIID complexes and plasmid DNA with or without the spacer insert were mixed in reaction buffer 3 (10 mM Tris pH 8.0, 50 mM KCl, 2 mM MgCl_2_) and incubated at 40°C for 20 min. The reaction was stopped by adding 1 μl of 20 mg/ml Proteinase K (Thermo scientific) and was further incubated at 37°C for 30 min. Then the reaction was phenol-chloroform (Ambion) extracted as described previously (32). Samples from the upper aqueous phase were mixed with formamide dye and resolved on 0.8% agarose gels. GeneRuler 1kb DNA ladder (Life Technologies) was used as a reference marker. Band intensities were quantified using ImageJ (37). Note that as EtBr may not bind supercoiled and relaxed DNA with equivalent binding densities, the absolute fraction of DNA cleaved is subject to some uncertainty. This does not affect the comparative analysis of wild-type and variant proteins.

## RESULTS

### Sso-IIID cleaves target RNA with 6 or 12 nt spacing

**Figure 1.**
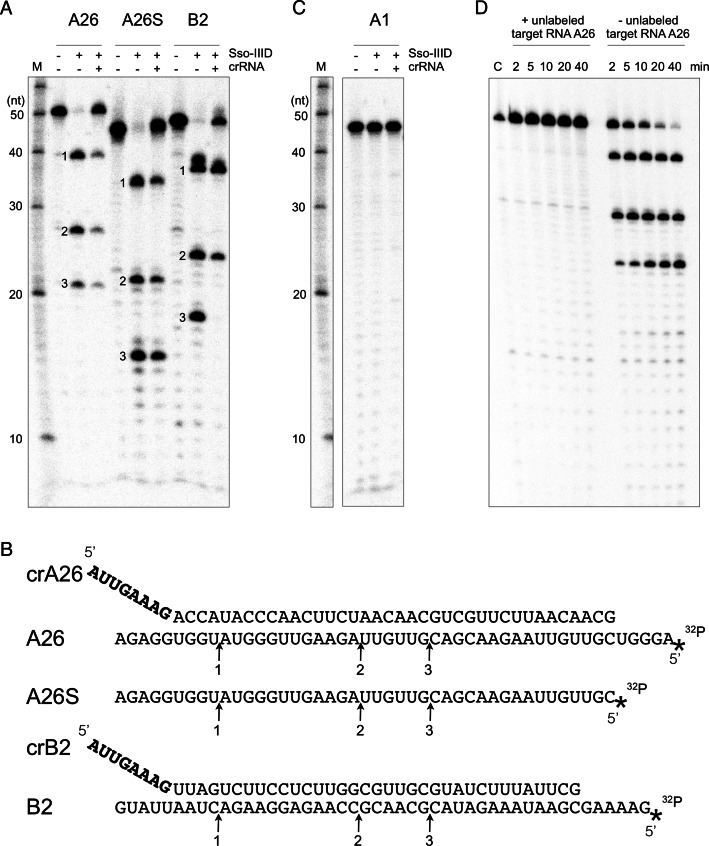
Sso-IIID cleaves target RNA with 6 or 12 nt spacing. 5′-^32^P end-labelled target RNA (4 nM) was incubated with purified endogenous Sso-IIID complex (500 nM) in the absence or presence of corresponding crRNA (100 nM) in reaction buffer at 75°C for 10 min, followed by denaturing gel electrophoresis and phosphorimaging. (**A**) Cleavage reactions with target RNA A26, A26S (a 5 nt 5′ truncation of A26) and B2. The three lanes for each target comprise target RNA alone, target RNA plus Sso-IIID, and target, Sso-IIID plus additional 100 nM crRNA complementary to the target. Major cleavage sites are labelled 1–3. **(B**) Sequences of the guide and target RNAs used in A. Arrows indicate the locations of the cleavage products 1–3. The 8 nt 5′-handle derived from the CRISPR repeat is shown in bold. (**C**) Cleavage reactions with target RNA A1, a sequence that is not represented in the crRNA content of purified Sso-IIID. The three lanes comprise target RNA alone, target RNA plus Sso-IIID, and target, Sso-IIID plus additional 100 nM crRNA complementary to the target. (**D**) Time course of cleavage of the A26 target RNA by Sso-IIID. The right hand lanes are for the standard reaction with 4 nM target. On the left, unlabelled target RNA to a final concentration of 10 μM was added prior to initiation of the assay. Other lanes: M—RNA ladder; C—Control lane with no added Sso-IIID.

The type IIIA complexes from *Streptococcus thermophilus, S. epidermidis* and *Thermus thermophilus* cleave target RNAs at regular 6 nt intervals that correspond to the spacing of the Cas7 backbone subunits (19,21,29). To test the Sso-IIID complex for this activity we incubated the complex with a labelled target RNA, A26, which has sequence complementary to one of the most abundant crRNAs previously identified after extraction from the Sso-IIID complex, comprising ≈5% of the total crRNA present (9). We observed three major cleavage products with a 12 or 6 nt spacing (Figure 1). Addition of further crRNA A26 reduced the activity, suggesting that the complex may not incorporate this crRNA productively *in vitro*. Deletion of 5 nt from the 5′ end of the target RNA (A26S) yielded the same 12–6 cleavage pattern with the fragments now 5 nt shorter, suggesting that cleavage is directed by a ruler mechanism from the 5′ end of the crRNA (Figure 1A), in common with other type III enzymes (19,23). The assay was repeated using a different target RNA, B2, which matches another abundant crRNA identified in the Sso-IIID complex (9). Again, a 12–6 nt spacing was observed and once again the addition of further cognate crRNA diminished the extent of target cleavage (Figure 1A). We proceeded to test Sso-IIID for backbone-mediated cleavage using a target RNA, A1, that is not present in the purified complex (9). No cleavage was observed, regardless of whether crRNA complementary to A1 was provided in the assay (Figure 1C).

Initial assays were performed in the presence of 2 mM Mg^2+^. Although no activity was detected in the presence of EDTA, a broad range of divalent metal ions supported backbone RNA cleavage by Sso-IIID, including Mg^2+^, Mn^2+^, Ca^2+^, Ni^2+^, Co^2+^ and Zn^2+^ (Supplementary Figure S1B). A similar broad specificity was observed for the *S. thermophilus* IIIA complex (19). To determine the 3′ end structure of the cleaved RNA species, we incubated the cleavage products with *E. coli* Polymerase A, which can extend RNA from a 3′-hydroxyl group. The cleavage products remained unchanged whereas the target RNA A26 was polyadenylated as expected (Supplementary Figure S1A). This is consistent with the presence of a 5′ hydroxyl group and a 2′,3′-cyclic phosphate moiety at the ends of the cleaved RNA products, as observed for other type III systems (13).

A time course of cleavage of the A26 target, performed under single turnover conditions where the Sso-IIID complex was present in molar excess (500 nM, of which 5% or 25 nM is loaded with crA26) over the substrate (4 nM), demonstrated very rapid cleavage of the target at sites 1–3 with ≈50% of substrate cleaved within the first 2 min. There was little evidence for interconversion between products, although the site 3 product built up more slowly over the course of the reaction, suggesting this site may be cleaved less rapidly than the others (Figure 1D). However, when a large excess (10 μM) of unlabelled target RNA A26 was added to the reaction, no substrate turnover was observed, even after 40 min. These observations suggest that backbone cleavage by Sso-IIID may be a single-turnover process, with product generation limited by the concentration of active enzyme present.

Together, these data show that the Sso-IIID complex shares the backbone-mediated RNA cleavage activity observed for several other type III systems, but with an unusual spacing that suggests one cleavage site is missed, which is explored further below. The Sso-IIID system is not ‘programmable’ with crRNA added *in trans*, suggesting that crRNA co-purifying with the complex is tightly bound and cannot be exchanged *in vitro*. Furthermore, this activity seems to be limited to a single-turnover, at least *in vitro*.

### Reconstitution of the Sso-IIID complex from individually expressed subunits

Each subunit of Sso-IIID was cloned and expressed individually, allowing reconstitution in recombinant form with a defined crRNA. The large subunit (Csm1, Sso1428) was expressed in *S. solfataricus* with a C-terminal polyhistidine tag, as described previously (9). Isolated Csm1 could be purified by metal affinity chromatography and separated from Csm1 in the Sso-IIID complex during size exclusion chromatography (Supplementary Figure S2). All of the other subunits were expressed in *E. coli* with cleavable N-terminal polyhistidine tags. Subunits Sso1424, 1425, 1426, 1430 and 1432 were obtained in soluble form and purified by metal affinity chromatography and size exclusion. Subunits Sso1427 and 1431 were insoluble after expression in *E. coli*. These proteins were purified by metal affinity chromatography in the presence of 8 M urea (Supplementary Figure S2). We reconstituted the Sso-IIID complex by mixing the individual subunits in the presence of a defined crRNA, as described in the methods. The reconstituted complex was purified by size exclusion chromatography, with the intact complex migrating as a peak corresponding to the retention time of the native complex, while sub-complexes and individual subunits eluted later (Supplementary Figure S2). Although inefficient in terms of yield, this approach provided sufficient active reconstituted complex for biochemical studies.

**Figure 2.**
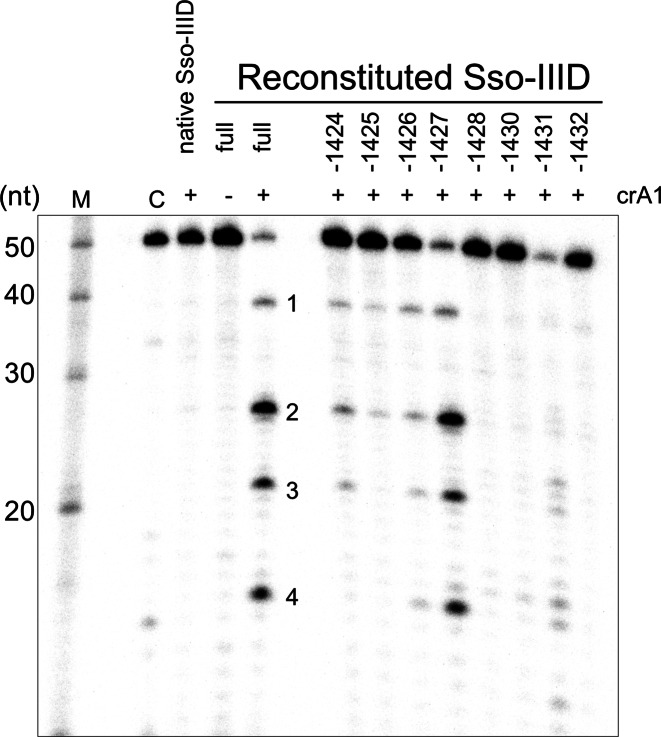
The Sso-IIID complex can be reconstituted in active form in vitro. 5′-^32^P end-labelled target RNA A1 (4 nM) was incubated with purified native Sso-IIID complex (500 nM) or recombinant, reconstituted Sso-IIID (rSso-IIID) in reaction buffer for 60 min at 75°C, followed by denaturing gel electrophoresis and phosphorimaging. Native Sso-IIID lacks crRNA A1 and thus does not cleave this target. Sso-IIID reconstituted from all the individual subunits (full complex) in the presence of crRNA A1 cleaves the A1 target RNA giving four products (1–4) with 12–6–6 spacing. Reconstitution experiments where one subunit was omitted (as indicated in the figure) showed varying levels of activity, with individual Cas7-like backbone subunits not essential for activity. Other lanes: M—RNA ladder; C—Control lane with no added Sso-IIID.

The Sso-IIID complex reconstituted with crRNA corresponding to the A1 spacer, which is not naturally present, catalysed cleavage of target RNA A1 with the same 12–6 nt spacing seen for the native complex (Figure 2). A further product (site 4) was observed, with a 6 nt spacing from site 3, suggesting that cleavage can occur at four different sites in the backbone. To investigate the subunit requirements of the complex, we reconstituted Sso-IIID with individual subunits omitted, one at a time. For these experiments, the reconstituted complex was not subjected to gel filtration. Reconstitutions that omitted the large subunit (Csm1/Sso1428), Sso1430, Sso1431 or Sso1432 all resulted in no detectable activity, suggesting that either complexes are not formed or are inactive when these subunits are absent. Sso1428 (Csm1/Cas10) and Sso1431 (Csm4/Cas5-like) form a close interaction at the base for all type III complexes (24,38,39). Native mass spectrometry places the Sso1430 subunit in contact with these two basal subunits in Sso-IIID (9), while Sso1432 is predicted to cap the Cas7-like backbone (9). When the small subunit (Cmr2/Sso1424) or the Cas7-like subunits Sso1425, or Sso1426 were missing, the cleavage activity was weakened but not eliminated, suggesting that these subunits play an important but not essential role. Omission of the Cas7-like subunit Sso1427 resulted in cleavage activity comparable to the full complex (Figure 2). For all these reconstituted complexes the cleavage pattern held the 12–6–6 pattern. These data suggest Sso-IIID has the potential to form a complex capable of backbone-mediated cleavage when one of the Cas7-like backbone subunits is absent, either by substituting the missing subunit with a paralogous one, or by forming sub-complexes that retain activity.

### Dual modes of RNA cleavage by the Sso-IIIB Cmr7-containing complex

**Figure 3.**
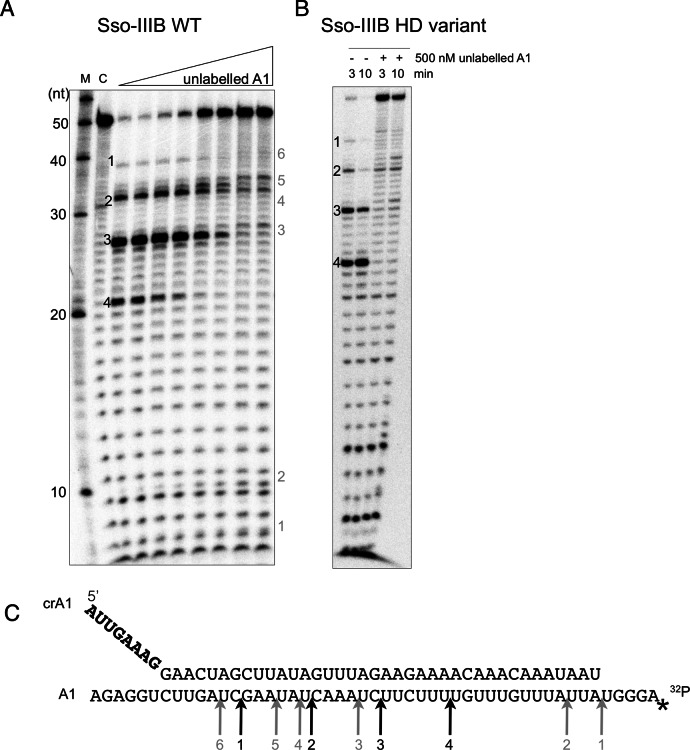
Dual modes of RNA cleavage by the Sso-IIIB complex. (**A**) 5′-^32^P end-labelled target RNA A1 (4 nM) was incubated with purified endogenous Sso-IIIB complex (500 nM) and crRNA A1 (100 nM) in reaction buffer, together with a varied concentration of unlabelled A1 target RNA (0–1000 nM). Reactions were incubated at 75°C for 20 min, followed by denaturing gel electrophoresis and phosphorimaging. Backbone-mediated cleavage with a 6–6–6 spacing pattern predominated where target concentration was low, but as total substrate concentration increased the cleavage mode switched to the UA cleavage observed previously. Major cleavage sites are labelled 1–4 in black for backbone cleavage and 1–6 in grey for UA cleavage. Other lanes: M—RNA ladder; C—A1 RNA incubated in absence of Sso-IIIB. (**B**) Backbone and UA cleavage modes for an Sso-IIIB complex with a variant HD nuclease domain (D181A/H182A) in the large (Cmr2) subunit. The HD variant retained strong backbone-mediated cleavage and significant UA cleavage activity. (**C**) Sequences of the guide and target A1 RNAs used in the above assay. Arrows indicate the locations of the cleavage products, labelled 1–4 in black for backbone cleavage and 1–6 in grey for UA cleavage.

The type IIIB complexes from *P. furiosus* and *T. thermophilius* have been demonstrated to cleave the target RNA with a regular 6 nt spacing pattern (11,40). In contrast with these studies, we previously reported that the Sso-IIIB complex, which includes the unique Cmr7 subunit, cleaves target RNA selectively at UA sites, rather than using a 6-nt ruler-based mechanism (14). One difference between our original report and the work of other labs was that we had used a large excess of target RNA over enzyme in our assays. To investigate this further, we carried out the RNA cleavage reaction with 500 nM Sso-IIIB and a fixed concentration of radioactively labelled target RNA (4 nM), and varied the total target RNA concentration by adding unlabelled target RNA, resulting in a final substrate range from 4 to 1000 nM (Figure 3). Consistent with our previous report, Sso-IIIB cleaved target RNA at UA sites when target RNA was present in excess of the enzyme (Figure 3A, right hand side). As cold target RNA was reduced below the concentration of Sso-IIIB, the pattern changed to the canonical backbone-mediated cleavage with 6 nt spacing observed for other type III effector complexes. Both cleavage patterns could be detected simultaneously at intermediate ratios.

To examine the possibility that the HD nuclease domain of the Sso-IIIB complex, which is conserved in all type III systems, played a role in the UA cleavage mode, we constructed a variant complex where the conserved ‘DH’ dipeptide in the HD domain was changed to an ‘AA’ motif. This variant was expressed in *S. solfataricus* and purified and assayed as for the wild-type protein (Figure 3B). The complex gave robust backbone-mediated cleavage of the A1 target RNA, as expected. The UA cleavage mode was still apparent, suggesting that the HD nuclease domain is not essential for this activity. We were unable to create variants in subunits such as Cmr4 or the unique Cmr7 subunit, as these are present in multi-copy in the complex and our experimental system does not allow deletion of the wild-type genes in *S. solfataricus*, with the result that complexes would contain mixtures of wild-type and variant subunits.

**Figure 4.**
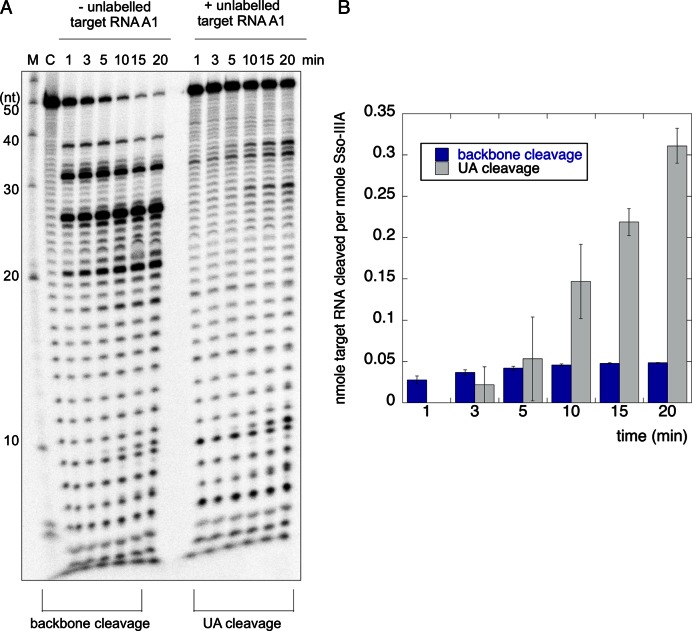
The two RNA cleavage modes of the Sso-IIIB complex possess distinct catalytic rates. (**A**) Time course reactions of backbone cleavage (left) and UA cleavage (right). Conditions were 40 nM Sso-IIIB complex with target RNA A1 at 2 nM (left), or 800 nM Sso-IIIB complex with total target RNA A1 at 500 nM (right). In both cases crRNA A1 was added to 100 nM final concentration. (**B**) Quantification of the extent of product formation for the two cleavage modes. The total nmoles of product formed per nmoles of protein is plotted against reaction time. Data points are the mean of triplicate experiments and standard errors are shown.

To further characterize the dual cleavage modes, we investigated the rate of substrate cleavage under the extreme conditions that result in only one cleavage mode (Figure 4). Under conditions of high enzyme excess, where only a single turnover is possible, backbone cleavage occurred very quickly, with ≈70% of the target RNA cleaved by the first time point at 1 min. There was little further conversion over the following 20 min reaction time course. These properties are consistent with those of Sso-IIID under similar conditions (Figure 1). To elicit the UA cleavage mode, the reaction was carried out under conditions of high substrate excess by adding unlabelled target RNA. Target RNA was cleaved in multiple turnover conditions as observed previously (14), with around 50% cut over the 20 min reaction. Both experiments were carried out in triplicate and gels were quantified by phosphorimaging. Figure 4B shows a plot of the amount of target RNA substrate cleaved per nmole of protein versus time for the two conditions. It is apparent that the backbone-mediated cleavage occurs in rapid, single turnover mode whilst UA cleavage displays initially slower kinetics that nonetheless result in considerably higher levels of substrate turnover over the course of the 20 min reaction time.

### DNA cleavage by the Sso-IIID complex

**Figure 5.**
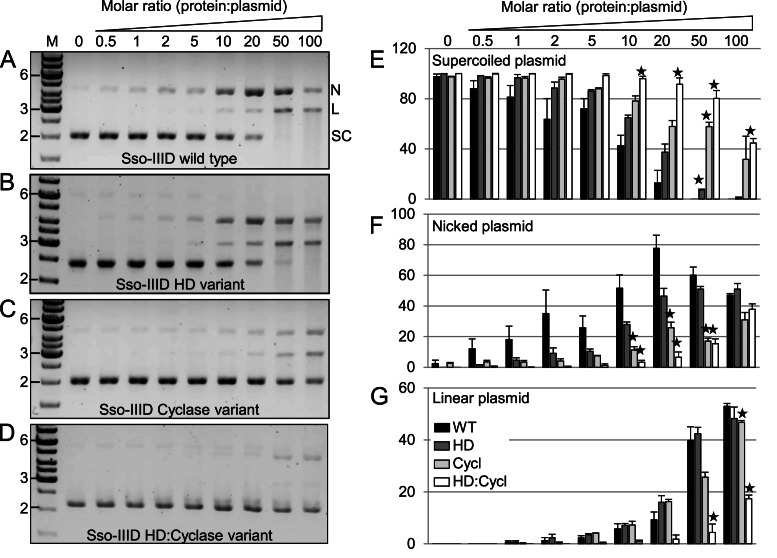
DNA cleavage by Sso-IIID is dependent on the HD and cyclase domains of the large subunit (Sso1428). Comparison of DNA cleavage activity between wild type (A) and three variant (B-D) Sso-IIID complexes. (**A**) The supercoiled DNA plasmid pSpacer (SC-bottom band) is nicked (N-top band) and linearized (L-middle band) by the wild type Sso-IIID complex, with no apparent supercoiled plasmid remaining at a 50:1 protein:plasmid ratio after a 20 min incubation. (**B–D**) The efficiency of nicking and linearization is slightly reduced in the HD variant (B), more significantly reduced in the cyclase variant (C), and severely impaired in the HD:cyclase double variant (D). (**E–G**) Reduction in cleavage activity for the three variants was quantified. Bars represent the mean band intensity for each plasmid species (where SC+L+N = 100) from triplicate experiments, and error is displayed as standard deviation (SD). Differences between the wild type and each variant Sso-IIID were determined using a T-test (one-tailed, unpaired), and significance is denoted by: ★ p< = 0.001. M- DNA marker (GeneRuler 1kb).

The activity of Sso-IIID wild type protein and three variants with amino acid replacements targeted to the HD domain, cyclase domain or both were tested for the ability to cleave plasmid DNA (Figure 5). We constructed a plasmid (pSpacer) containing sequences matching those of abundant crRNAs present in the Sso-IIID and –IIIB complexes. Supercoiled pSpacer plasmid DNA was cleaved by the wild type protein to generate nicked and linear DNA products (Figure 5A). This activity did not depend on the presence of a cognate target DNA sequence within the plasmid, as the parental plasmid was cleaved similarly (not shown). This suggests that the activity is unregulated compared to that expected *in vivo*. Single-strand nicking occurs first, with the generation of increasing quantities of linear DNA over time, suggesting that nicked products are further converted into linear ones by a second strand cleavage activity.

To investigate this further we compared the activities of variants with targeted changes in the HD and cyclase active sites, quantifying the proportions of supercoiled, nicked and linear DNA as a function of protein:DNA ratio in replicate experiments. The HD variant showed a modest but discernible defect in processing of supercoiled DNA compared to the wild type complex, with a small reduction in the amounts of nicked products generated (Figure 5B). The cyclase variant gave a stronger defect, with significantly more uncleaved plasmid apparent at the highest protein concentrations. The double variant yielded the most profound defect, with a significant proportion of the DNA uncleaved at the highest protein concentrations and strongly reduced generation of nicked or linear products, even when compared to the cyclase variant (Figure 5D, G). These data suggest that both the HD and cyclase domains play a role in DNA cleavage in Sso-IIID. It should be noted that, particularly for high protein:DNA concentrations, this assay may not necessarily discriminate between the independent nicking of both strands due to two distinct Sso-IIID complexes and the coordinate nicking of both strands by the two active sites of a single complex. This may explain why the single HD and cyclase variants can still generate some linear DNA.

## DISCUSSION

### Backbone-mediated RNA cleavage by Sso-IIID and IIIB

The observation that the Sso-IIID and IIIB-Cmr7 complexes are both capable of target RNA cleavage with a backbone-mediated 5′ ruler mechanism means that all type III systems tested i*n vitro*, including these two divergent examples, possess this type of activity. Although the early EM studies of Sso-IIIB reported a ‘crab claw’ structure (14), the biochemical data presented here support the presence of a canonical Cas7 backbone found in all Type I and Type III complexes. For Sso-IIIB, the four RNA cleavage sites probably correspond to the positions of the Cmr4 (Sso1987) subunits that make up the backbone of many type IIIB complexes (22). As previously reported (19) the Sso1987 subunit has a conserved aspartate residue (D34) equivalent to D33 in the *S. thermophilus* type IIIA and D26 in the *P. furiosus* IIIB systems, both of which are essential for backbone cleavage activity (19,22). For technical reasons, we could not generate a variant form of Sso-IIIB uniformly mutated at this position, as gene knockouts are problematic in *S. solfataricus*.

**Figure 6.**
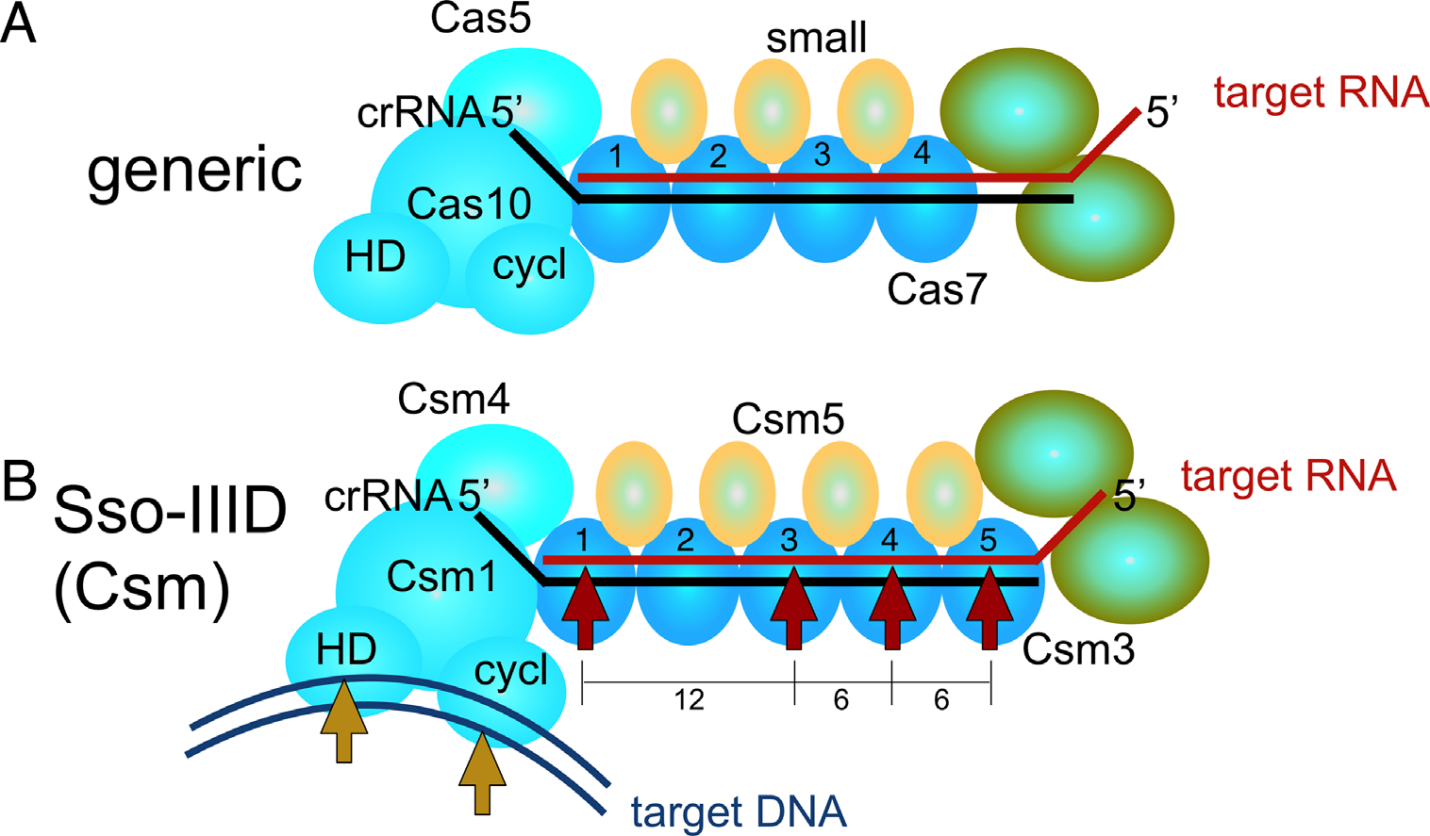
Schematic representation of the subunit organisation and target nucleic acid cleavage patterns for type III complexes. (**A**) Organisation of a generic type III complex. The Cas10-Cas5 sub-complex defines the ‘foot’ of the structure and binds the 5′ end of the crRNA. The Cas10 subunit has two nuclease active sites: HD nuclease (HD) and cyclase (cycl). The backbone is made up of a variable number of Cas7 and small subunits, whilst the ‘head’ has one or two subunits (green), which are not always essential. The crRNA is shown in black and target RNA in red. (**B**) The Sso-IIID complex has a five-subunit backbone composed of four copies of Sso1426 and one of Sso1425. Cleavage of the target RNA (red arrows) is observed with a 12–6–6 spacing, consistent with cutting at sites 1, 3, 4 and 5. For clarity, subunits whose role and placement are unclear are omitted from this diagram. Target DNA (magenta) may be cleaved by the HD and cyclase nuclease sites of the Csm1 subunit on separate strands (orange arrows).

For the Sso-IIID effector the situation is more complex. Figure 6 shows a schematic representation of a generic type III complex, with details for Sso-IIID shown below. The 12–6–6 nt spacing observed for three different target RNA species suggests that one specific position in the backbone does not support RNA cleavage. Sso-IIID has two closely related Cas7-like subunits, Sso1425 and Sso1426, which are thought to be present at a stoichiometry of 1 and 4, respectively (9). A simple explanation for the missed cleavage site would therefore be that the single Sso1425 subunit does not support target RNA cleavage. However, reconstituted Sso-IIID complexes lacking either Sso1425 or Sso1426 both support target RNA cleavage with the same 12–6–6 spacing as the native protein (Figure 2). This suggests that considerable plasticity in the backbone composition of Sso-IIID is possible, and also rules out the simple explanation of one inactive subunit for the missed cleavage site. An alternative explanation is that the crRNA:target RNA duplex adopts a different conformation at the position corresponding to the missed site, which is at position 2 in the backbone (Figure 6). This would explain the conservation of the 12–6–6 spacing with different target RNAs and different subunit compositions. Given that the Sso-IIID complex has more distinct subunits than any other type III system (eight, as opposed to seven in Sso-IIIB and six in most other type III complexes), it is perhaps not surprising that there may be subtle differences in RNA binding. Indeed, the Sso1426 subunit lacks the highly conserved ‘D33’ residue identified as responsible for backbone- mediated cleavage in the *S. thermophilus* type IIIA complex (19).

One possibility is that backbone-mediated RNA cleavage, which has largely been detected *in vitro* and which predominates under conditions of enzyme excess, may not be a relevant activity *in vivo*. In support of this, the abolition of backbone-mediated RNA cleavage in the *S. epidermidis* type IIIA system did not significantly affect plasmid immunity *in vivo*, whilst abrogation of DNA targeting abolished immunity (29). Backbone-mediated RNA cleavage by the *S. thermophilus* type IIIA system has been shown to provide resistance against infection by the ssRNA phage MS2 *in vivo* (19), but it is open to question whether this phage is a relevant target for CRISPR systems as it is unclear how protospacers could be captured from such a virus.

*Sulfolobus islandicus REY15A*, encodes two type III systems. One resembles the Sso-IIIB Cmr7 complex and does not appear to target DNA *in vivo*. The other is a more canonical type IIIB system and has been demonstrated to possess both RNA and transcription-dependent DNA targeting that provides immunity against phage. The activities of these two complexes have been investigated *in vivo*. Both could target mRNA when programmed with a cognate crRNA sequence. The complex without Cmr7 was shown to cleave RNA with a 6 nt ruler mechanism, whilst the complex with Cmr7 cleaved at UA sites, and the latter complex provided more efficient RNA targeting *in vivo* (16). These data suggest that the UA cleavage mechanism of Sso-IIIB and related complexes, which predominates *in vitro* when RNA targets are present in excess, may be the dominant activity *in vivo*. Unfortunately, the active site responsible for UA cleavage remains to be determined. Mutation of the HD nuclease active site generated a variant protein that retained UA cleavage activity (Figure 3), whilst backbone-mediated cleavage activity was unaffected. The Cmr7 subunit is an attractive candidate for a catalytic role in UA cleavage. Unfortunately Cmr7 is present at a high stoichiometry in the Sso-IIIB complex (14) and we are unable to express and purify Sso-IIIB variants lacking wild-type copies of the Cmr7 subunit as knockouts are technically very difficult in the *Sulfolobus* strain used for these experiments.

### DNA targeting activities of type III complexes

A variety of nuclease activities have been ascribed to type III effector complexes (Figure 6). In addition to a presumably universal backbone-mediated RNA cleavage mechanism, distinct nuclease activities have been ascribed to the large subunit of type III systems. The *Thermococcus onnurineus* Cas10 subunit acts as a metal dependent nuclease *in vitro* with both circular and linear ssDNA substrates and the activity appears to be located in the HD domain (18). In contrast the DNA nuclease activity of *S. epidermidis* Cas10 *in vitro* was located in the cyclase domain (41), in keeping with recent studies showing cyclase-mediated cleavage of DNA during transcription (29). Here we have shown that the intact Sso-IIID complex also cleaves plasmid DNA *in vitro*, albeit in a sequence-independent manner that may represent a deregulated aspect of its activity. It appears that the cyclase domain is more important for plasmid nicking than the HD domain, however the most striking observation is that both the cyclase and HD domain active sites are required for efficient conversion of supercoiled DNA into linear form. This raises the prospect that these two nuclease domains in the Cas10 subunit each cut one strand of target DNA duplexes during interference *in vivo* (Figure 6). This would be analogous to the situation in Cas9, where there are two nuclease active sites, targeting opposing strands (42). The hypothesis that both the HD and cyclase domains, which are conserved and function as nucleases under certain circumstances, collaborate to cleave target DNA duplexes *in vivo*, is an attractive one. However, this awaits definitive evidence. Clearly, there is still much to learn about the activities and functions of the type III CRISPR effector complexes.

## Supplementary Material

SUPPLEMENTARY DATA
