# Supplementary material for: Multiple nucleic acid cleavage modes in divergent type III CRISPR systems
Source: Nucleic Acids Res. 2016 Jan 21;44(4):1789–99. doi: 10.1093/nar/gkw020 (PMC4770243; doi:10.1093/nar/gkw020)
Supplement: SUPPLEMENTARY DATA [file supp_44_4_1789__index.html]

Multiple nucleic acid cleavage modes in divergent type III CRISPR systems — SUPPLEMENTARY DATA 

# Multiple nucleic acid cleavage modes in divergent type III CRISPR systems

## SUPPLEMENTARY DATA

- SUPPLEMENTARY DATA
