# Supplementary material for: Multiple nucleic acid cleavage modes in divergent type III CRISPR systems
Source: Nucleic Acids Res. 2016 Jan 21;44(4):1789–99. doi: 10.1093/nar/gkw020 (PMC4770243; doi:10.1093/nar/gkw020)
Supplement: SUPPLEMENTARY DATA [file supp_gkw020_nar-03160-h-2015-File008.pdf]

Supplementary data for **Multiple nucleic acid cleavage modes in divergent type III CRISPR systems**

Jing Zhang, Shirley Graham, Agnes Tello , Huanting Liu and Malcolm F White*

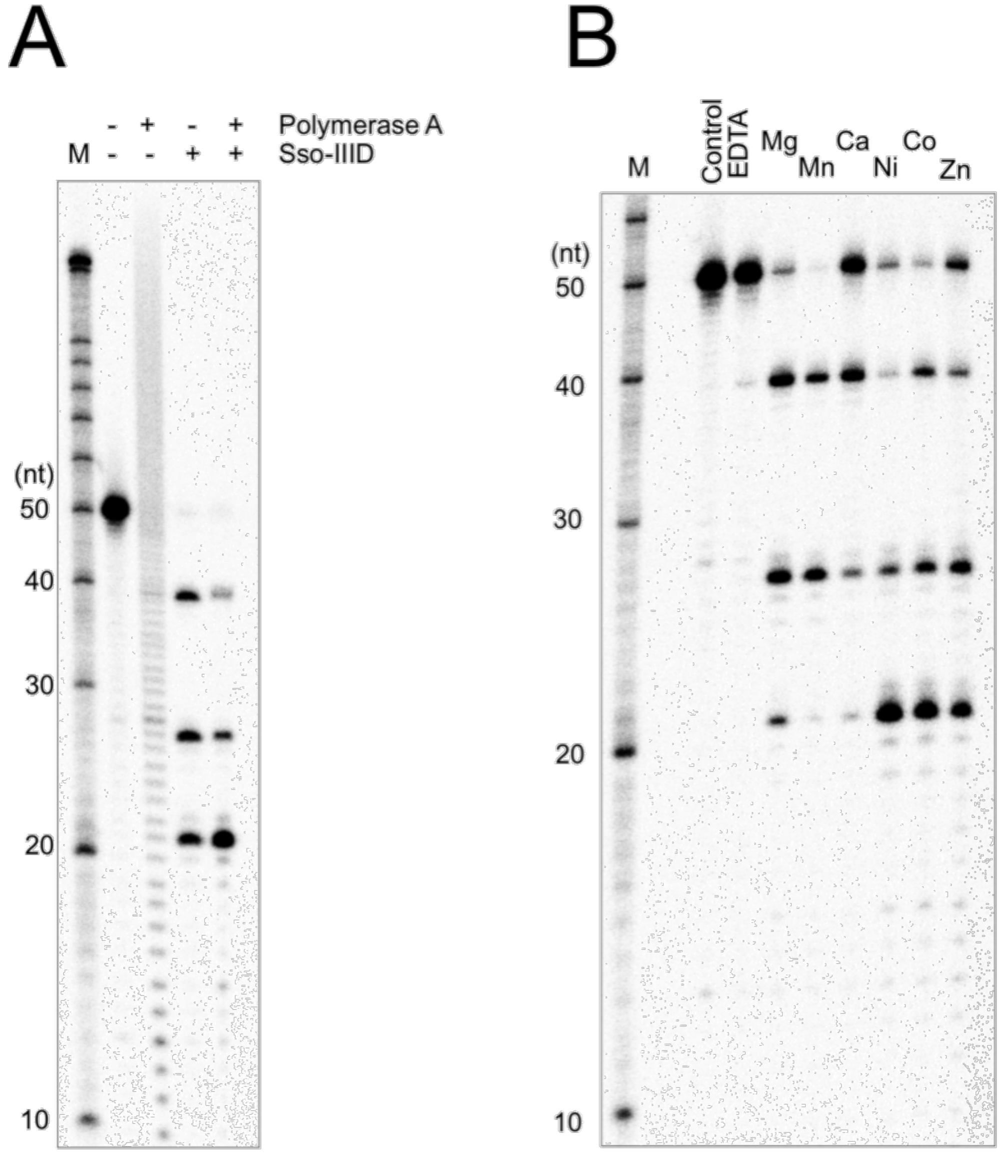

Supplementary figure 1

A. The cleavage products of the Sso-IIID complex lack 3' hydroxyl groups. 5' end-labelled target RNA A26 and its Sso-IIID complex cleavage products were subjected to polyadenylation by *E. coli* Polymerase A for 30 min at 37 °C.

B. Various divalent metals can support the cleavage activity of the Sso-IIID complex. RNA cleavage reactions were performed in the presence of either EDTA (2 mM) or the indicated divalent metals (2 mM).

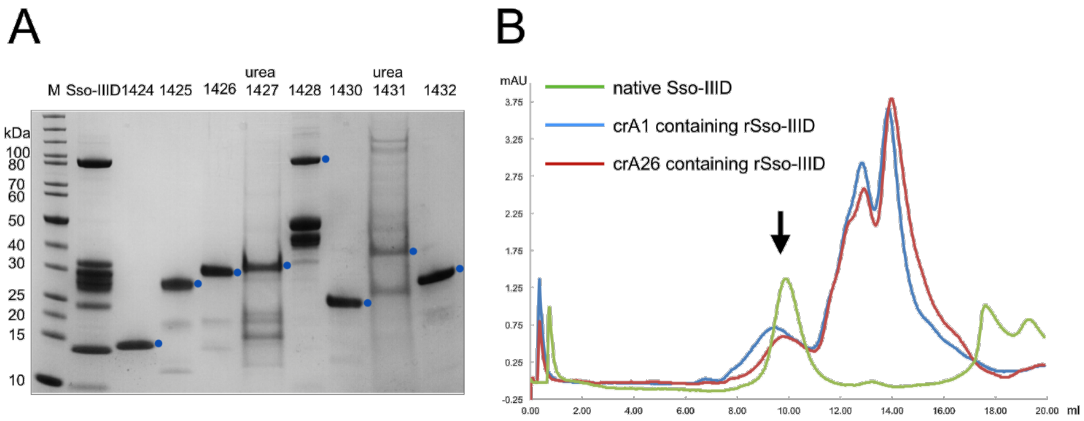

Supplementary figure 2

A. SDS-PAGE analysis of isolated subunits of Sso-IIID used in the reconstitution of the complex. The expected subunit position in the gel is indicated with a dot. The large (Csm1 / Sso1428) subunit degraded partially on storage, giving rise to the two small bands seen. The Sso1427 and Sso1431 subunits were purified in the presence of 8 M urea and refolded in the presence of the other subunits plus crRNA.

B. Size-exclusion chromatography of the reconstituted Sso-IIID complex and the endogenous complex. The green line indicates the UV elution trace of an endogenous Sso-IIID complex. The blue line indicates the trace of the Sso-IIID complex reconstituted with crA1 and red line with crA26. Both have a peak (arrowed) corresponding to the expected size for the intact complex and significant absorbance due to sub-complexes and individual subunits eluting at later times.

Sequence of the gBlock for plasmid cleavage assays:

5'-GGACTGGATC CCGTAGCTCA GGCCTCTGCG CCCTTGAGAC CATACCCAAC TTCTAACAAC GTCGTTCTTA ACAACGGTGA AGGTCGGTGT GAACGGGTGA GTTCCAGGGC TTAGTCTTCC TCTTGGCGTT GCGTATCTTT ATTCGGGTGC TGAGTATGTC GTGGAGTCTA CTGGTGTCTT TCGTATAAGG ACCAGAACGG CAATACCCAA ACTGTGTCGA CTGGTATGTA GGCAGTGGGG AGGGATCCAA CGC
